# Effect of Fe_3_O_4_ Nanoparticles Modified by Citric and Oleic Acids on the Physicochemical and Magnetic Properties of Hybrid Electrospun P(VDF-TrFE) Scaffolds

**DOI:** 10.3390/polym15143135

**Published:** 2023-07-24

**Authors:** Vladimir Botvin, Anastasia Fetisova, Yulia Mukhortova, Dmitry Wagner, Sergey Kazantsev, Maria Surmeneva, Andrei Kholkin, Roman Surmenev

**Affiliations:** 1 International Research & Development Center “Piezo- and Magnetoelectric Materials”, Research School of Chemistry & Applied Biomedical Sciences, National Research Tomsk Polytechnic University, 634050 Tomsk, Russia; 2 Physical Materials Science and Composite Materials Center, Research School of Chemistry & Applied Biomedical Sciences, National Research Tomsk Polytechnic University, 634050 Tomsk, Russia; 3 Scientific Laboratory for Terahertz Research, National Research Tomsk State University, 634050 Tomsk, Russia; 4 Institute of Strength Physics and Materials Science of Siberian Branch of Russian Academy of Sciences, 634055 Tomsk, Russia; 5 School of Natural Sciences and Mathematics, Ural Federal University, 620000 Ekaterinburg, Russia

**Keywords:** P(VDF-TrFE), magnetite, electrospinning, modification, magnetoactive scaffolds

## Abstract

This study considers a fabrication of magnetoactive scaffolds based on a copolymer of vinylidene fluoride and trifluoroethylene (P(VDF-TrFE)) and 5, 10, and 15 wt.% of magnetite (Fe_3_O_4_) nanoparticles modified with citric (CA) and oleic (OA) acids by solution electrospinning. The synthesized Fe_3_O_4_-CA and Fe_3_O_4_-OA nanoparticles are similar in particle size and phase composition, but differ in zeta potential values and magnetic properties. Pure P(VDF-TrFE) scaffolds as well as composites with Fe_3_O_4_-CA and Fe_3_O_4_-OA nanoparticles demonstrate beads-free 1 μm fibers. According to scanning electron (SEM) and transmission electron (TEM) microscopy, fabricated P(VDF-TrFE) scaffolds filled with CA-modified Fe_3_O_4_ nanoparticles have a more homogeneous distribution of magnetic filler due to both the high stabilization ability of CA molecules and the affinity of Fe_3_O_4_-CA nanoparticles to the solvent used and P(VDF-TrFE) functional groups. The phase composition of pure and composite scaffolds includes a predominant piezoelectric β-phase, and a γ-phase, to a lesser extent. When adding Fe_3_O_4_-CA and Fe_3_O_4_-OA nanoparticles, there was no significant decrease in the degree of crystallinity of the P(VDF-TrFE), which, on the contrary, increased up to 76% in the case of composite scaffolds loaded with 15 wt.% of the magnetic fillers. Magnetic properties, mainly saturation magnetization (*M*_s_), are in a good agreement with the content of Fe_3_O_4_ nanoparticles and show, among the known magnetoactive PVDF or P(VDF-TrFE) scaffolds, the highest *M*_s_ value, equal to 10.0 emu/g in the case of P(VDF-TrFE) composite with 15 wt.% of Fe_3_O_4_-CA nanoparticles.

## 1. Introduction

Piezo-, magnetoactive and magnetoelectric materials, due to their unique properties, are of considerable interest to material scientists [1]. Such materials are widely used in medicine [2], pharmaceuticals [3], microelectronics [4], the manufacture of modern smart devices [5], textiles [6], and other promising areas. From the point of view of practical application, magnetoelectric materials exhibiting a magnetoelectric effect occupy a special place [7]. The fabrication of such materials is of key importance for modern regenerative medicine and tissue engineering, since it allows the development of high-tech devices and treatment techniques and their implementation in medical practice, improving the quality of care provided.

Currently known magnetoelectric materials are mainly inorganic single-phase compounds (Bi_5_Ti_3_FeO_15_, LiFe_5_O_8_, etc.) and composites based on ferromagnetic materials (metals—Co, Ni, alloys—FeCo, FeGa, Metglas, Terfenol-D, NiFe_2_O_4_, CoFe_2_O_4_, etc.) and piezoelectric ceramics (PbZr_x_Ti_(1−x)_O_3_, BaTiO_3_, AlN, etc.) [8]. These materials, despite the high values of their magnetoelectric and piezoelectric coefficients, have a number of disadvantages in terms of biocompatibility, mechanical, and other properties, which limit their use and implementation in biomedicine. To eliminate the mentioned drawbacks, it was proposed to use composites, including a piezopolymer matrix and a nanosized magnetic filler [9]. Among polymers, poly(vinylidene fluoride) (PVDF) and a copolymer of vinylidene fluoride with trifluoroethylene (P(VDF-TrFE)) have the highest piezoelectric properties due to the presence of a piezoactive β-phase (TTTT-conformation), along with other phases with lower piezoelectric properties (γ-phase—TTTGTTTG′ conformation and δ-phase—TGTG′ conformation) [10]. PVDF and P(VDF-TrFE) can also exist at a non-piezoelectric α- (TGTG′ conformation) and ε-phase (TTTGTTTG′ conformation) [10]. The formation of certain P(VDF-TrFE) phases depends on the thermodynamic conditions and the methods of materials synthesis [10]. Therefore, the stabilization of the piezoactive β-phase in PVDF as well as in PVDF-based copolymers is a significant challenge. In the case of P(VDF-TrFE), in contrast to PVDF, the β-phase is stabilized due to a steric hindrance caused by the presence of bulky trifluoroethylene repeating units of the copolymer [11]. Magnetite (Fe_3_O_4_) nanoparticles can act as a magnetic filler of the magnetoelectric composites since they are quite easily synthesized, undergo the necessary surface modification, have a high saturation magnetization, and are biocompatible [12].

The method of obtaining P(VDF-TrFE)- or PVDF-based materials also affects the formation of the β-phase. The most suitable method for the fabrication of magnetoactive materials is electrospinning, because it contributes to the stabilization of the β-phase as a result of in situ poling [13] and makes it possible to obtain materials with a given morphology and physicochemical properties [14]. It is known that Fe_3_O_4_ nanoparticles are prone to agglomeration, which affects the size of nanoparticles and other properties, including magnetic ones [12]. To reduce the agglomeration of Fe_3_O_4_ nanoparticles during the synthesis and upon fabrication of PVDF or P(VDF-TrFE) composites, a surface of the magnetic filler is functionalized with various organic and high-molecular-weight modifiers, such as mono- and polyfunctional carboxylic acids [15,16], poly(N-vinylpyrrolidone) [17], xanthan gum [18], poly(dopamine) [19], and others. The distribution of magnetic nanoparticles in the electrospun PVDF or P(VDF-TrFE) fibers requires comprehensive study, since it can affect many physicochemical and operational properties of materials. However, there are a small number of the studies considering features of magnetic nanoparticles distribution in the electrospun P(VDF-TrFE) (PVDF) fibers loaded, as a rule, with unmodified Fe_3_O_4_ nanoparticles [20,21]. The addition of unmodified and modified Fe_3_O_4_ nanoparticles as well as other magnetic fillers to PVDF or P(VDF-TrFE) can also affect the degree of crystallinity of the polymer, the content of the piezoactive β-phase, and consequently the piezoelectric properties of the resulting materials [22]. Depending on the concentration and chemical nature of the surface of the magnetic fillers, both an increase and a decrease in the degree of crystallinity and the content of the β-phase can be observed. In this regard, when developing magnetoactive piezoelectric materials, the influence of both factors should be taken into account. An analysis of known studies has shown that to date there are no studies on the effect of Fe_3_O_4_ nanoparticles functionalized with modifiers of various natures on the physicochemical, magnetic, and other properties of P(VDF-TrFE)-based materials. To assess the effect of functional groups on the surface of Fe_3_O_4_ nanoparticles and the nature of their interactions with P(VDF-TrFE) functional groups, we proposed to use nanoparticles modified with hydrophobic and hydrophilic modifiers, for example, with citric and oleic acids.

Previously, we had obtained magnetoactive P(VDF-TrFE) scaffolds of various compositions filled with Fe_3_O_4_ nanoparticles modified with oleic acid (OA) demonstrating hydrophobic properties [23]. Considering the nature of the functional groups of the P(VDF-TrFE), it is of considerable interest to fabricate and study composite magnetoactive scaffolds with modifiers, which, on the one hand, exhibit hydrophilic and hydrophobic properties, and, on the other hand, reduce the tendency of Fe_3_O_4_ nanoparticles to agglomerate. Citric acid (CA) and OA can act as hydrophilic and hydrophobic modifiers, respectively. Thus, the present research is devoted to the fabrication of electrospun magnetoactive scaffolds based on P(VDF-TrFE) and Fe_3_O_4_ nanoparticles functionalized with hydrophilic and hydrophobic modifiers and a comprehensive study of their structure, morphology, particle distribution, and physicochemical properties.

## 2. Materials and Methods

### 2.1. Materials

The powder of P(VDF-TrFE) (70/30) (MW = 300,000 Solvene 300/P300), FeCl_3_·6H_2_O, and FeSO_4_∙7H_2_O were purchased from Sigma Aldrich (Steinheim, Germany). Dimethylformamide (DMF), acetone, ammonia solution, CA, and OA were purchased from local vendors and used without further purification.

### 2.2. Synthesis of Magnetite Nanoparticles Modified by Citric and Oleic Acids

**Figure 1:**
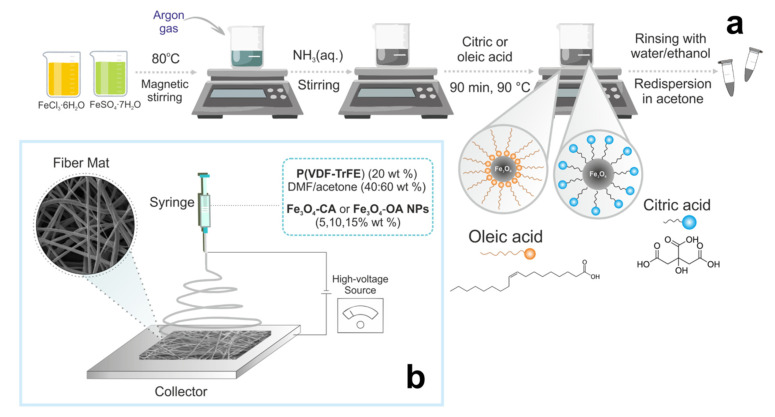
Scheme of the synthesis of modified Fe_3_O_4_ nanoparticles (**a**) and fabrication of magnetoactive P(VDF-TrFE) scaffolds by electrospinning (**b**).

Synthesis of magnetite nanoparticles modified by citric (Fe_3_O_4_-CA) and oleic (Fe_3_O_4_-OA) acids was carried out according to the previously reported method (Figure 1a) [24]. Briefly, FeCl_3_∙6H_2_O (3.32 g) and FeSO_4_∙7H_2_O (1.22 g) were dissolved in 200 mL of deionized water under an argon atmosphere by magnetic stirrer (1300 rpm) and heated to 80 °C. Then, 20 mL of ammonia solution was added and stirring was continued for an additional 5 min. Thereafter, 1.4 mL of CA or OA solution (0.5 g/mL) was added to the reaction mixture and synthesis was carried out for another 90 min at 90 °C. At the end of the synthesis, thus-obtained Fe_3_O_4_-CA or Fe_3_O_4_-OA nanoparticles were separated by the magnet, washed with water and ethanol until neutral pH was achieved, and then used as a suspension in acetone. The concentration of synthesized modified nanoparticles in suspension was determined by the gravimetry method [23].

### 2.3. Preparation of Electrospinning Solutions

The calculated amount of P(VDF-TrFE) was dissolved in a mixture of DMF: acetone (40:60 wt.%) to obtain polymer solutions with the concentration of 20 wt.% suitable for the fabrication of scaffolds with a fiber diameter of about 1 μm (Figure 1b). The solution was mixed using a shaker at room temperature overnight until the formation of a clear solution.

In the case of composite scaffolds with Fe_3_O_4_-CA and Fe_3_O_4_-OA concentrations of 5, 10, and, 15 wt.% (relative to polymer weight), the calculated volume of DMF and the portion of magnetite suspension in acetone were mixed with P(VDF-TrFE) powder in a shaker overnight.

### 2.4. Electrospinning Parameters

Pure P(VDF-TrFE) and composite scaffolds doped with Fe_3_O_4_-CA nanoparticles were fabricated by a custom-made electrospinning setup. The prepared polymer solution was loaded into a 5-mL plastic syringe with a stainless-steel needle (inner diameter 21 gauge). The flow rate of the solution was regulated by a syringe pump (Aitecs 2016 Universal). Polymer and composite fibers were deposited on the cylindrical rotating collector. All the samples were obtained using the following parameters: distance between the needle and the collector of 13 cm, applied voltage of 15.0 kV (15.5 kV in the case of composite scaffold obtainment), solution flow rate of 0.2 mL∙h^−1^, and collector rotation speed of 200 rpm.

### 2.5. Characterization of the Samples

Fourier transform infrared (FTIR) spectra were recorded using a Cary 630 FTIR spectrometer (Agilent Technologies, Santa Clara, CA, USA) in the wavelength range 4000–400 cm^−1^. IR spectra of Fe_3_O_4_-CA and Fe_3_O_4_-OA nanoparticles were recorded in absorbance mode (mixing with KBr and subsequent formation of thin tablets). Spectra of pure P(VDF-TrFE) and composite scaffolds were acquired as received in an attenuated total reflectance (ATR) mode (diamond crystal).

The phase compositions of Fe_3_O_4_-CA, Fe_3_O_4_-OA nanoparticles, P(VDF-TrFE), and composite scaffolds were analyzed by X-ray diffraction (XRD) on a MiniFlex 600 diffractometer (Rigaku corporation, Tokyo, Japan) at scattering angles ranging from 10 to 70° (CuKα radiation). The crystallite size was calculated using Scherrer’s equation (Equation (1)).

(1) $$D=\frac{k\lambda }{\beta cos\theta },$$

where *k* is the proportionality constant, *λ* is the X-ray wavelength (nm), *β* is the enlargement of the measured diffraction line at the mid-height of its maximum intensity (in radian units), and *θ* is the XRD peak position.

Raman spectra Fe_3_O_4_-CA and Fe_3_O_4_-OA nanoparticles were recorded on an NT-MDT system (NT-MDT Spectrum Instruments, Zelenograd, Russia) equipped with a 100× objective. Excitation was performed with a laser at wavelengths of 633 nm with a maximum power of 60 mW. To prevent heating and oxidation of magnetite, no more than 1% of the laser power was used during measurements.

The thermal properties of pure and composite scaffolds were studied on STA 449 F1 (Netzsch, Selb, Germany) in the range of 25–300 °C in an argon atmosphere at a heating rate of 10 °C/min. The crystallinity (*X*_c_) of the polymer and composite scaffolds was evaluated using Equation (2):

(2) $$X_{c}=\frac{{\Delta H}_{f}}{\left(1-\omega \right){\Delta H}_{f}^{0}}\cdot 100\%,$$

where ω is a weight fraction of Fe_3_O_4_-CA and Fe_3_O_4_-OA in composite scaffolds, and the heat of fusion for 100% crystalline P(VDF-TrFE) with VDF:TrFE ratio of 70:30 mol.% (${\Delta H}_{f}^{0}$) equaling 45 J∙g^−1^ [25].

The morphology of Fe_3_O_4_-CA, Fe_3_O_4_-OA nanoparticles and pure and composite P(VDF-TrFE) scaffolds was studied by scanning electron microscopy (SEM) with Apreo 2 SEM electron microscope (Thermo Fisher, Tokyo, Japan).

Distribution of modified Fe_3_O_4_ nanoparticles in the P(VDF-TrFE) fibers was studied by transmission electron microscopy (TEM) on a JEM-2100 electron microscope (JEOL, Tokyo, Japan). To prepare samples for TEM measurements, a fragment of the scaffolds was fixed using Araldide epoxy resin, and then layers of 80–120 nm thickness were cut using ultratome «Ultrotome III» («LKB», Stockholm, Sweden).

The zeta potential of Fe_3_O_4_-CA and Fe_3_O_4_-OA nanoparticles was determined from their electrophoretic mobility in a U-shaped cell with gold electrodes with front fixation by dynamic light scattering on a ZetaSizer Nano Zs device (Malvern, Worcestershire, UK).

The magnetic properties of Fe_3_O_4_-CA, Fe_3_O_4_-OA nanoparticles and composite scaffolds were investigated at 300 K with an external pulsed magnetic field of up to 6.5 kOe using a pulsed magnetometer (Tomsk State University, Tomsk, Russia). The technical characteristics of the magnetometer and the technique for measuring the magnetic properties are described in detail in [26].

## 3. Results and Discussion

**Figure 2:**
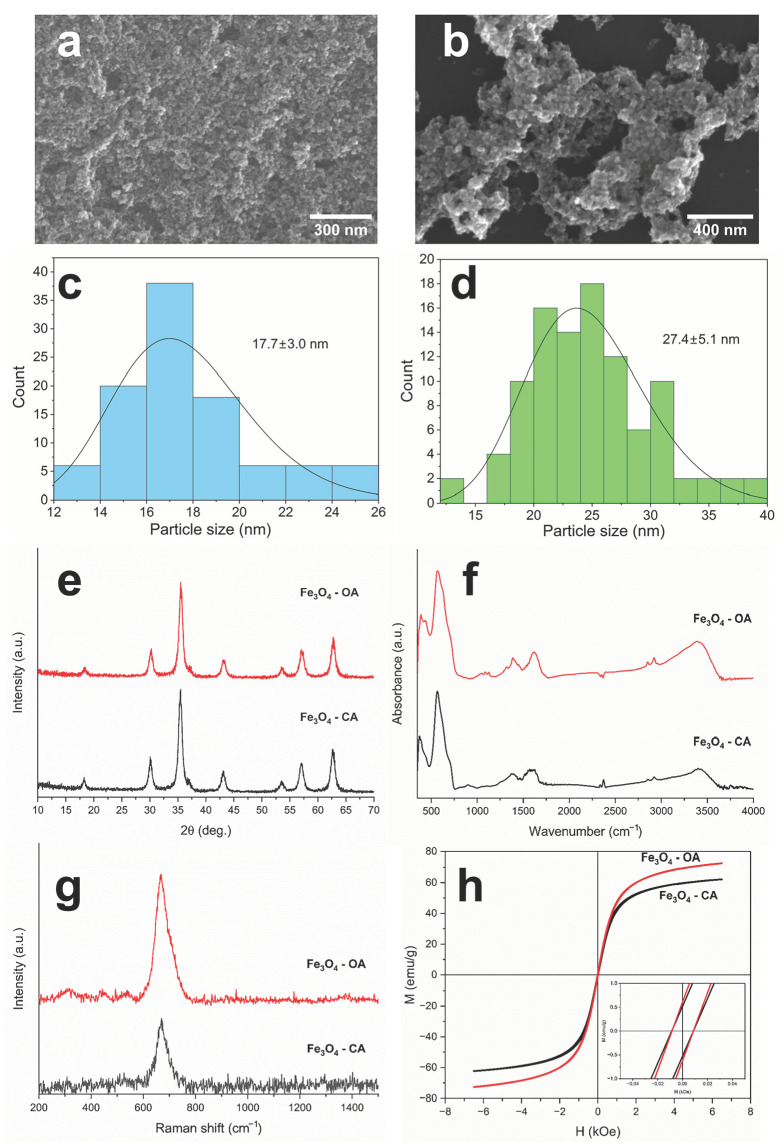
SEM images (**a**,**b**) and size distribution (**c**,**d**) of Fe_3_O_4_-CA (**a**,**c**) and Fe_3_O_4_-OA (**b**,**d**) nanoparticles, their XRD patterns (**e**), IR (**f**) and Raman (**g**) spectra, and magnetic hysteresis loops (**h**).

Synthesis conditions have a significant influence on the morphology, phase composition, and, subsequently, magnetic characteristics of Fe_3_O_4_ nanoparticles [12]. Due to features of a physicochemical nature, unmodified Fe_3_O_4_ nanoparticles are prone to agglomeration, which affects their particle size. In order to reduce the agglomeration of nanoparticles during synthesis, various groups of modifiers are used [27]. CA and OA were chosen as modifiers, since they should result in a decrease in the agglomeration of nanoparticles during their synthesis, and subsequently when obtaining magnetoactive P(VDF-TrFE) scaffolds [15,16]. SEM micrographs of Fe_3_O_4_-CA and Fe_3_O_4_-OA nanoparticles (Figure 2a,b) showed a uniform size distribution of the particles without any visible agglomerates, which confirms the influence of carboxylic acid modifiers. The average sizes of Fe_3_O_4_-CA and Fe_3_O_4_-OA nanoparticles were 17.7 and 24.7 nm, respectively (Figure 2c,d).

XRD patterns of both types of nanoparticles (Figure 2e) contained characteristic reflections of Fe_3_O_4_ at 2θ = 18.23°, 30.0°, 35.36°, 37.0°, 42.99°, 53.37°, 56.87°, and 62.45° corresponding to d_hkl_ crystal planes at (111), (220), (311), (222), (400), (422), (511), and (440), respectively [28]. The crystallite sizes of Fe_3_O_4_-CA and Fe_3_O_4_-OA nanoparticles calculated by Scherrer’s equation (Equation (1)) are 11.6 and 13.0 nm.

IR spectroscopy makes it possible to establish not only the absorption bands of Fe_3_O_4_ nanoparticles, but also the absorption bands of modifier acids. Thus, the IR spectra of Fe_3_O_4_-CA and Fe_3_O_4_-OA (Figure 2f) contain, along with the absorption band related to the vibrations of the Fe–O bond of magnetite at 568 cm^−1^, the absorption bands corresponding to the vibrations of the functional groups of CA and OA in the regions 3000–2950, 1607, and 1372 cm^−1^, which are related to the C–H stretching vibrations, symmetric and asymmetric vibrations of the carboxylate anion of the modifier acids, respectively [29,30]. The presence of a band corresponding to the carboxylate anion confirms the binding of carboxyl groups of CA and OA onto the surface of magnetite nanoparticles via chemisorption. Raman spectra of modified Fe_3_O_4_ nanoparticles (Figure 2g) supplement data regarding chemical and phase composition. Both spectra contain characteristic peaks at 302, 539, and 670 cm^−1,^ related to magnetite [31]. In the case of Fe_3_O_4_-OA, the Raman spectrum additionally demonstrates a low-intensity shoulder at 700 cm^−1^, which corresponds to the maghemite phase (γ-Fe_2_O_3_). The last could have formed both during magnetite synthesis and due to the effect of a laser beam during Raman measurements [23]. The crystal structures of Fe_3_O_4_ and γ-Fe_2_O_3_ have similar crystal lattice parameters; therefore, it is difficult to distinguish them by XRD [32]. Thus, a comprehensive study of Fe_3_O_4_ nanoparticles by XRD, IR, and Raman spectroscopy led to a determination of a real phase composition of magnetite nanoparticles and confirmed the presence of modifier acids on their surface.

Modification of Fe_3_O_4_ nanoparticles with CA and OA differed in their functionality, leading to a change in the properties of the nanoparticle surface. Such modification should affect the distribution and stability of nanoparticles in the P(VDF-TrFE) solution as well as the nature of the polymer–magnetic filler interactions. The measured values of the zeta potential for Fe_3_O_4_-CA and Fe_3_O_4_-OA turned out to be −35.3 and −2.6 mV, respectively, which is consistent with the functionality of modifier acids [33,34]. The CA molecules located on the surface of magnetite nanoparticles have two free carboxyl groups capable of ionization, defining the measured values of the zeta potential [34]. In contrast to the CA structure, the OA molecule includes a polar carboxyl group and a nonpolar long alkyl substituent, therefore acting as a surfactant and promoting the formation of micelle-like structures upon interaction with the surface of oxide nanoparticles [35]. Depending on the OA concentration, a single- or a double-layer structure can be formed during the functionalization of the Fe_3_O_4_ surface, affecting the physicochemical properties, including the zeta potential value [33]. At a low concentration of OA, single-layer structures are predominantly formed, as in the case of our Fe_3_O_4_-OA nanoparticles. This is consistent with the measured zeta potential, which is slightly negative.

Modification of the surfaces of Fe_3_O_4_ nanoparticles also naturally results in a change of magnetic properties, which are important in the synthesis of the magnetoactive polymer composite scaffolds based on them. Figure 2h shows magnetic hysteresis loops of Fe_3_O_4_-CA and Fe_3_O_4_-OA nanoparticles, and their main magnetic characteristics are presented in Table 1. The saturation magnetization (*M*_s_) of Fe_3_O_4_-CA and Fe_3_O_4_-OA nanoparticles was 61.9 and 72.6 emu/g, which is inferior to the *M*_s_ value of bulk unmodified magnetite [36]. The decrease in *M*_s_ in the case of modified Fe_3_O_4_ nanoparticles is caused by the small sizes of the particles. A surface layer with unordered magnetic spins is found on them, and they convert to a paramagnetic state at room temperature [37,38]. The decrease in *M*_s_ is also associated with the content in the non-magnetic layer of the modifier acid in the samples under study [39]. Higher *M*_s,_ in the case of Fe_3_O_4_-OA, is caused by a lower concentration of OA on the nanoparticle surface. The presence of only one carboxyl group of OA compared to the three carboxyl groups of CA, and the good complexation ability of citrates in general, leads to a more effective binding of CA molecules to the Fe_3_O_4_ nanoparticle surface. This additionally confirms the formation of a single OA layer on the surface of Fe_3_O_4_ nanoparticles. Our previous study of Fe_3_O_4_-OA nanoparticles demonstrates that according to thermogravimetric measurements, a weight loss equal to the amount of OA is nearly of 6% [23]. The thermogravimetric curve of Fe_3_O_4_-CA nanoparticles shows weight loss of about of 13% [40]. In addition, a more significant decrease in the magnetization of Fe_3_O_4_-CA nanoparticles can also be associated with a smaller crystallite size than that of Fe_3_O_4_-OA, which leads to an increase in the previously mentioned surface layer. The coercive forces (*H*_c_) for both types of nanoparticles have close to non-zero values, which characterizes them as ferromagnets. Such materials are capable of magnetization reversal in weak magnetic fields.

**Figure 3:**
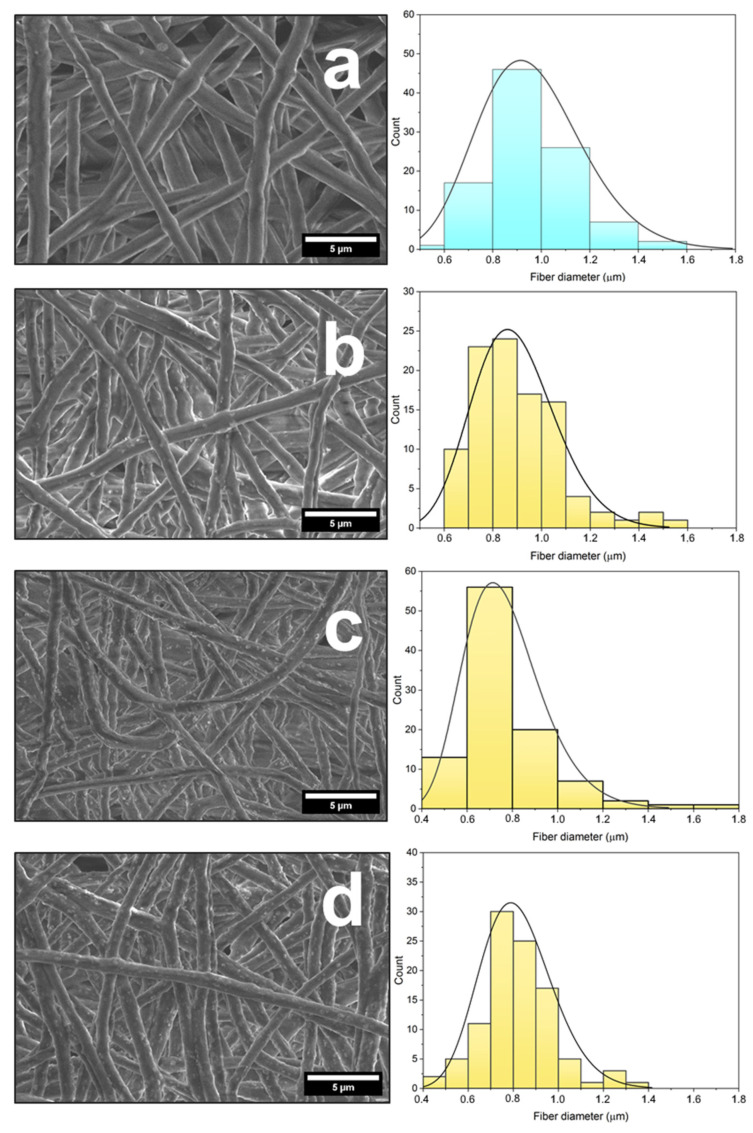

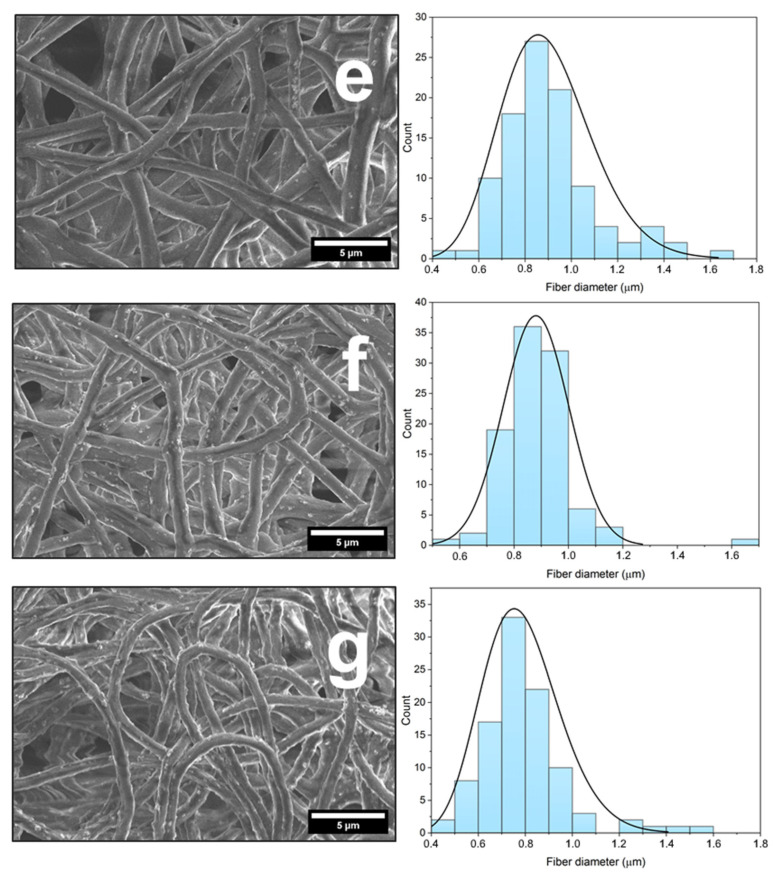
SEM images and fiber diameter distributions of pure P(VDF-TrFE) (**a**) and composite scaffolds filled with 5, 10, and 15 wt.% of Fe_3_O_4_-CA (**b**–**d**) and Fe_3_O_4_-OA (**e**–**g**) nanoparticles.

A fabrication of magnetoactive scaffolds based on P(VDF-TrFE) and Fe_3_O_4_ nanoparticles by electrospinning facilitates the formation of materials with controlled properties. However, electrospinning proceeding at high voltages in a medium of various organic solvents, as a rule, results in changes in morphology, phase composition, and other physicochemical properties of all components of the scaffolds. When obtaining electrospun composite scaffolds filled with Fe_3_O_4_ nanoparticles, a special attention should be paid to the morphology and distribution of the magnetic filler in the P(VDF-TrFE) fibers. To obtain defects-free fibers, optimally selected parameters of the electrospinning, including the appropriate solvent, should be chosen [39]. Figure 3 shows SEM micrographs and the fiber diameter distributions of pure P(VDF-TrFE) and composite scaffolds filled with different contents of Fe_3_O_4_-CA and Fe_3_O_4_-OA nanoparticles.

The fabricated scaffolds do not contain any defects and have an average fiber diameter of about 1 µm. The known studies indicate that such a fiber diameter should have a positive effect on human cells in the biomedical application of the developed materials, since it is comparable to the fiber diameters of many tissues. [40]. The SEM micrographs of composite scaffolds, as well as the diameters of the obtained fibers, allow us to evaluate the distribution of filler nanoparticles on their surface. Regardless of the magnetic filler type, the surface of composite scaffolds contains Fe_3_O_4_ nanoparticles, whose amount increases with increasing concentration. When comparing the distribution of Fe_3_O_4_ nanoparticles modified with CA and OA on the surfaces of composite fibers, P(VDF-TrFE)/Fe_3_O_4_-CA scaffolds demonstrate a more uniform distribution without formation of large agglomerates. Such observations indicate that CA molecules have a greater stabilizing effect on Fe_3_O_4_ nanoparticles, and the modified nanoparticles themselves have a greater affinity to P(VDF-TrFE) macromolecules. Together, CA molecules prevent an agglomeration of Fe_3_O_4_-CA nanoparticles and contribute to the most consistent distribution of the nanoparticles in the P(VDF-TrFE) fibers. Despite the presence of nanoparticles on the surface of P(VDF-TrFE) fibers, all of them are coated with a polymer layer. Previously, we observed such an effect for magnetoactive polymer scaffolds based on unmodified Fe_3_O_4_ nanoparticles [41]. When P(VDF-TrFE) solution in the mixture of DMF and acetone is stretched into a polymer jet under an electric field and deposited on a collector, dispersed Fe_3_O_4_ nanoparticles remain covered with a polymer layer in the fiber due to surface tension forces.

**Figure 4:**
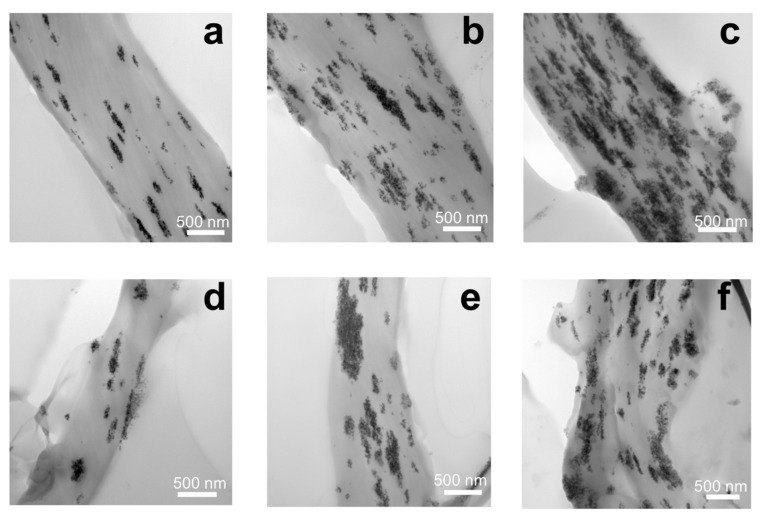
TEM images of P(VDF-TrFE) composite scaffolds filled with 5, 10, and 15 wt.% of Fe_3_O_4_-CA (**a**–**c**) and Fe_3_O_4_-OA (**d**–**f**) nanoparticles.

The properties of magnetoactive composite scaffolds are certainly affected by the distribution of magnetic filler nanoparticles, not only on the surfaces of P(VDF-TrFE) fibers, but also in the bulk. Figure 4 shows TEM micrographs of P(VDF-TrFE) composite scaffolds filled with Fe_3_O_4_-CA and Fe_3_O_4_-OA nanoparticles. The analysis of the micrographs indicates that in the case of P(VDF-TrFE)/Fe_3_O_4_-CA scaffolds (Figure 4a–c), a uniform distribution of the filler is observed in proportion to a given concentration, which is not accompanied by the formation of large agglomerates. The resulting small agglomerates have a morphology elongated in the direction of the flow and deposition of the polymer solution jet during electrospinning. P(VDF-TrFE)/Fe_3_O_4_-OA scaffolds (Figure 4d–f) are characterized by a less uniform distribution of the particles due to the nature of the particles themselves, the solvents used, and the way they were introduced into the electrospinning solution.

One of the factors affecting the uniform distribution of modified Fe_3_O_4_ nanoparticles, mainly with CA molecules, is the introduction of a magnetic filler into the P(VDF-TrFE) solution as a suspension. The suspension method prevents agglomeration of Fe_3_O_4_ nanoparticles, which is not always possible when a magnetic filler is added to a solution as a dry powder [41]. Another factor affecting the uniform distribution of Fe_3_O_4_ nanoparticles is the presence of modifier carboxylic acids on their surface. For example, the presence of free carboxyl groups on the surfaces of Fe_3_O_4_ nanoparticles modified with CA prevents their agglomeration due to an electrostatic repulsion [34]. Moreover, the polar carboxyl groups of Fe_3_O_4_-CA nanoparticles have a high affinity to the polar solvents (DMF, acetone) used in the polymer solution, as well as to the functional groups of P(VDF-TrFE). In the case of Fe_3_O_4_-OA nanoparticles, agglomeration can decrease both due to a steric hindrance caused by the bulky OA alkyl moiety [33] and hydrophobic interactions with low-polar groups of hydrophobic P(VDF-TrFE). However, the greatest stabilizing effect (decrease of agglomeration) of both factors in the fabrication of P(VDF-TrFE) composite scaffolds has been observed when Fe_3_O_4_ nanoparticles were modified with CA, which was confirmed by SEM and TEM results.

**Figure 5:**
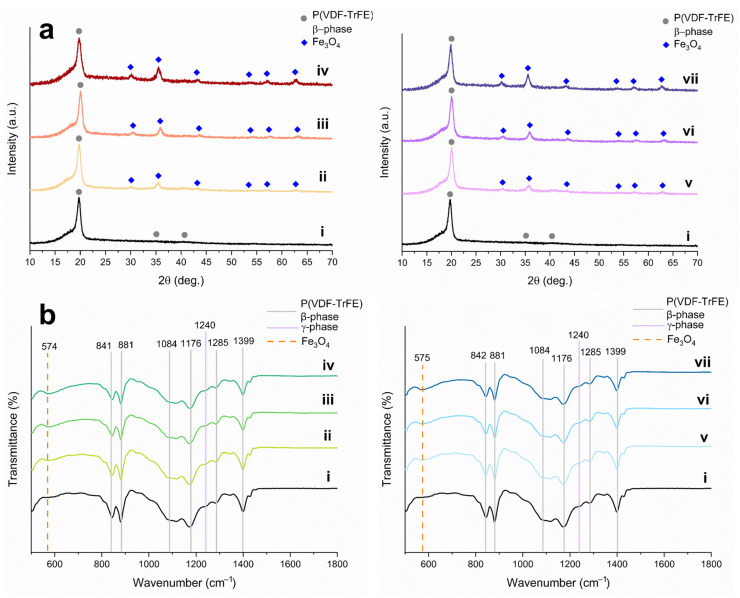
XRD patterns (**a**) and IR spectra (**b**) of pure P(VDF-TrFE) (i) and composite scaffolds filled with 5, 10, 15 wt.% of Fe_3_O_4_-CA (ii–iv) and Fe_3_O_4_-OA (v–vii) nanoparticles.

A phase composition of polymeric and inorganic components of magnetoactive scaffolds is of great importance, since they significantly affect physicochemical and, subsequently, operational properties. The phase composition of P(VDF-TrFE) correlates with a piezoresponse, whereas the phase composition of Fe_3_O_4_ nanoparticles is associated with magnetic properties, mainly with saturation magnetization. Figure 5 presents diffraction patterns of pure and composite P(VDF-TrFE) scaffolds filled with Fe_3_O_4_-CA and Fe_3_O_4_-OA nanoparticles of various content.

The XRD pattern (Figure 5a,i) of pure P(VDF-TrFE) scaffolding includes reflections of the β-phase at 19.8°, 35.3°, and 41.0°, related to the crystallographic planes (110)/(200), (001), and (201), respectively [23]. There is also a low-intensity shoulder at 18.3°, indicating the contribution of the (020) crystal plane of the monoclinic γ-phase, which has less pronounced piezoelectric properties [10]. At the same time, the characteristic reflections of the P(VDF-TrFE) α-phase at 17.6°, 19.9°, and 26.6° are absent in the XRD pattern [10]. XRD patterns of composite scaffolds (Figure 5a, ii–vii), along with reflections of the β- and γ-phases of P(VDF-TrFE), demonstrate reflections of the Fe_3_O_4_ phase with an intensity proportional to the concentration of the magnetic filler. The absence of side reflections of other iron oxides and oxyhydroxide phases indicates that electrospinning does not affect the phase composition of the magnetic filler of the magnetoactive scaffolds.

For a more detailed study of the phase composition and conformation of pure P(VDF-TrFE) and composite scaffolds filled with Fe_3_O_4_-CA and Fe_3_O_4_-OA nanoparticles, the fabricated scaffolds were studied by IR spectroscopy. This method allows analysis of the macromolecules’ conformation, determining a qualitative composition and, for some systems, the quantitative content of the P(VDF-TrFE) phases [42]. IR spectra of pure P(VDF-TrFE) scaffolds and composites with Fe_3_O_4_-CA and Fe_3_O_4_-OA nanoparticles are presented in Figure 5b.

The IR spectra in the range from 500 to 1800 cm^−1^ contain characteristic absorption bands at 842, 881, 1084, 1176, 1285, and 1399 cm^−1^, related to the crystalline β-phase of P(VDF-TrFE), mainly to vinylidene fluoride repeating units [43,44]. The band at 1399 cm^−1^ indicates bending vibrations of CH_2_ groups and stretching vibrations of C–C groups of P(VDF-TrFE), the bands at 1283 and 1176 cm^−1^ correspond to symmetric and asymmetric stretching vibrations of CF_2_ groups, and the bands at 880 cm^−1^ and 841 cm^−1^ refer to rocking bending vibrations of CH_2_ groups and asymmetric and symmetric stretching vibrations of CF_2_ groups, respectively [44]. The band at 1116 cm^−1^ refers to the characteristic vibrations of the trifluoroethylene units of the copolymer, along with a low-intensity band at 1341 cm^−1^, which belongs to the bending vibrations of C–H in the CHF group [45]. A detailed analysis of IR spectra demonstrates an absence of characteristic absorption bands of the P(VDF-TrFE) α-phase at 762, 976, and 1207 cm^−1^ [45]. This observation proves the role of the trifluoroethylene repeating units of the copolymer in the stabilization of the β-phase.

The IR spectra exhibit, additionally, a low-intensity band at 1240 cm^−1^, which belongs to a weaker piezoelectric γ-phase of the P(VDF-TrFE) [46]. Another characteristic band of the γ-phase at 840 cm^−1^ coincides with one of the bands of the β-phase, which makes it less informative. The formation of the γ-phase during the electrospinning of PVDF and its derivatives was also noted elsewhere [47,48,49]. Such a phase transition occurs due to the influence of the electric field [47] and the nature of solvents used, as well as their ratio [50], on the crystallization of P(VDF-TrFE) during electrospinning. Mentioned factors lead to a partial transformation of the TTTT conformation of the β-phase to the TTTGTTTG′ of the γ-phase conformation. A reliable quantitative calculation of the phase composition in P(VDF-TrFE) scaffolds in comparison with PVDF is difficult, because, on the one hand, unlike the α-phase, most of the bands of the β- and γ-phases are superimposed due to the similarity of conformation of their repeating units [51]. On the other hand, one of the characteristic bands of the γ-phase at 1240 cm^−1^ is present in the spectrum as a low-intensive shoulder, which complicates the baseline correction and subsequent calculation [10]. The spectra of composite scaffolds also contain a band observed at 574 cm^−1^, which refers to the Fe–O stretching vibrations of Fe_3_O_4_ nanoparticles [40]. The intensity of this band naturally increases with an increase in the concentration of the magnetic filler in the scaffolds.

**Figure 6:**
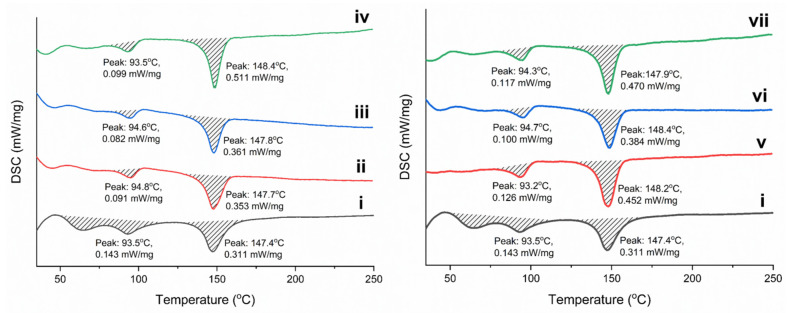
DSC curves of pure P(VDF-TrFE) (i) and composite scaffolds filled with 5, 10, 15 wt.% of Fe_3_O_4_-CA (ii–iv) and Fe_3_O_4_-OA (v–vii) nanoparticles.

It is known that the incorporation of nanoparticles into polymer scaffolds results in a change in thermal properties and crystallization behavior. The latter is important in assessing the degree of crystallinity of the polymer, which is consistent with the content of the β-phase and the piezoelectric properties of P(VDF-TrFE). To study the effect of modified Fe_3_O_4_ nanoparticles on the thermal properties and degree of crystallinity, fabricated scaffolds were studied by DSC. Figure 6 demonstrates the DSC curves of pure and composite P(VDF-TrFE) scaffolds, and Table 2 includes the main thermal characteristics and the degree of crystallinity.

All DSC curves contain an endo peak in the region of 93.1–94.8 °C, which refers to the P(VDF-TrFE) phase transition from the ferroelectric to the paraelectric state (Curie transition). Such a temperature of transition is characteristic of a copolymer with a ratio of VDF:TrFE units of 70:30 mol.% [52]. The melting temperature, which is characterized by the second endo peak on the DSC curves, is in the region of 147.4–148.4 °C. A slight increase in the melting temperature in the case of composite scaffolds is due to the influence of the magnetic filler on the melting of P(VDF-TrFE) crystallites. The degree of crystallinity of the fabricate scaffolds has an unusual behavior. In a comparison of both types of nanoparticles, it can be noticed that the degree of crystallinity varies nonmonotonically, especially in the case of composite scaffolds filled with Fe_3_O_4_-OA nanoparticles. Such regularity is in agreement with a less uniform distribution of Fe_3_O_4_-OA nanoparticles in the fibers, which was confirmed by SEM and TEM. With an increase in the concentration of the magnetic filler, no significant decrease in the degree of crystallinity is observed. Moreover, it is important to note that for composite scaffolds containing 15 wt.% of both types of Fe_3_O_4_ nanoparticles, the highest value of the degree of crystallinity of about 76% is observed. This result is a consequence of the interactions of CA and OA located on the surface of Fe_3_O_4_ nanoparticles with functional groups of P(VDF-TrFE) macromolecules promoting their crystallization. An increase in the degree of crystallinity of magnetoactive composite scaffolds can have a positive effect on the piezoelectric properties of materials, which depend both on the total crystallinity and the content of the β-phase.

**Figure 7:**
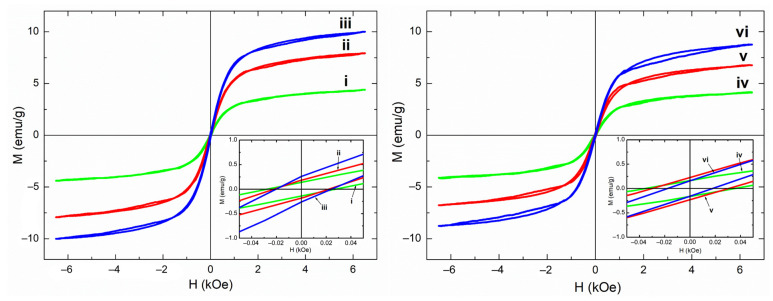
Hysteresis loops of magnetoactive P(VDF-TrFE) scaffolds filled with 5, 10, 15 wt.% of Fe_3_O_4_-CA (i–iii) and Fe_3_O_4_-OA (iv–vi) nanoparticles.

A chemical nature of the modifier, the phase composition and distribution of the magnetic filler in the polymer fibers of composite scaffolds, has a significant effect on their magnetic properties. Figure 7 shows the magnetic hysteresis loops of P(VDF-TrFE) composite scaffolds with different contents of Fe_3_O_4_-CA and Fe_3_O_4_-OA nanoparticles, and Table 3 lists their main magnetic characteristics.

With an increase in the concentrations of both types of Fe_3_O_4_ nanoparticles in the composition of the scaffolds, a regular increase in *M*_s_ is observed. Thus, for P(VDF-TrFE) scaffolds with 5, 10, and 15 wt.% of Fe_3_O_4_-CA nanoparticles, the *M*_s_ values were 4.4, 7.9, and 10.0 emu/g, respectively. For scaffolds with similar concentrations of Fe_3_O_4_-OA nanoparticles, *M*_s_ was 4.2, 6.8, 8.8 emu/g. All samples of composite scaffolds have a non-zero *H*_c_, which characterizes them as ferromagnetic materials. The increase in the *H*_c_ of all composite scaffolds is associated with a low content of magnetite particles. The particles cease to interact magnetostatically with each other; as a result, magnetite chain aggregates disrupt in the composite [53]. The value of *H*_c_ facilitates a distribution of magnetic nanoparticles in the polymer fibers. For P(VDF-TrFE) scaffolds with 5 and 10 wt.% Fe_3_O_4_-OA nanoparticles, large *H*_c_ values can be observed, which indicates a less uniform distribution of filler particles and the formation of agglomerates. This is in agreement with the results of SEM, TEM, and DSC. The lower affinity of P(VDF-TrFE), solvents, and Fe_3_O_4_-OA nanoparticles can also explain a decrease in *M*_s_ of composite scaffolds as being due to the lower concentration of the filler than theoretically calculated values. It is important to note that the achieved *M*_s_ for P(VDF-TrFE) scaffolds with Fe_3_O_4_-CA nanoparticles, to the best of our knowledge, has the highest value among known PVDF or P(VDF-TrFE) composite scaffolds, considering the introduction of a high concentration of magnetic filler and its uniform distribution in the polymer fibers.

**Figure 8:**
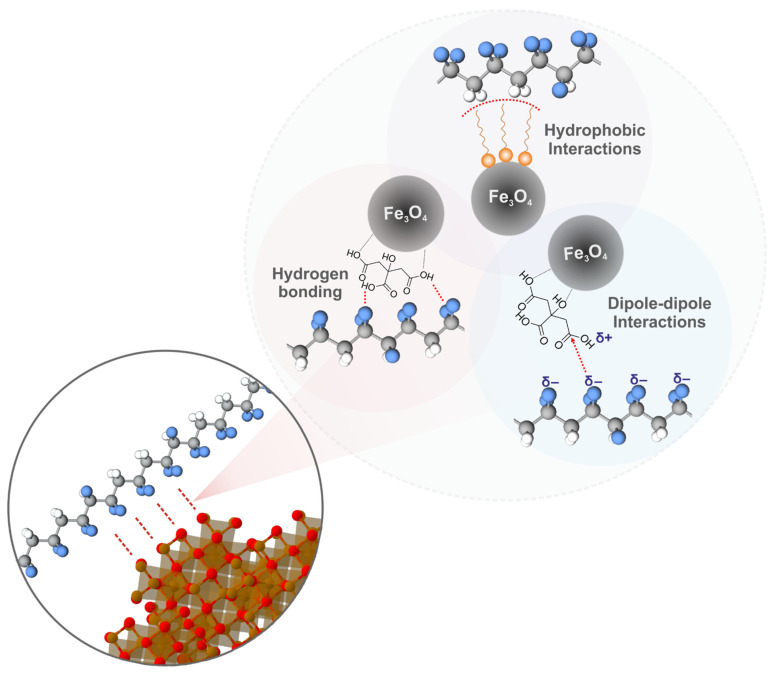
Proposed scheme of interactions of P(VDF-TrFE) and Fe_3_O_4_ nanoparticles modified with CA and OA.

Taking into account the results of studying the morphology, phase composition, and physicochemical properties of P(VDF-TrFE) scaffolds with Fe_3_O_4_ nanoparticles modified with CA and OA, the possible types of polymer–magnetic filler interactions leading to a high degree of affinity are shown in Figure 8.

The most probable type of interaction is intermolecular hydrogen bonding of highly electronegative fluorine atoms of P(VDF-TrFE) and hydrogen atoms of carboxylic acid modifiers [54]. The presence of polarized carboxyl groups on the surface of modified Fe_3_O_4_ nanoparticles allows them to enter into dipolar interactions with dipoles of P(VDF-TrFE) macromolecules. A similar interaction mechanism was proposed for PVDF composites with organic molecules and polymers containing polarized functional groups [55,56]. In the case of Fe_3_O_4_-OA nanoparticles, as already noted, hydrophobic interactions of the long alkyl OA substituent with low-polarity P(VDF-TrFE) moieties are possible [57]. It should be noted that such types of interaction can have a lesser effect on P(VDF-TrFE) macromolecules, since the β-phase in them is stabilized by bulky trifluoroethylene repeating units, but, at the same time, they can significantly affect the α → β phase transitions in PVDF macromolecules, resulting in an improvement of the piezoelectric properties of piezoactive polymer.

## 4. Conclusions

Magnetoactive P(VDF-TrFE) composite scaffolds filled with 5, 10, and 15 wt.% of Fe_3_O_4_ nanoparticles modified with CA and OA were fabricated by electrospinning. The phase compositions of the modified Fe_3_O_4_ nanoparticles were represented by a pure magnetite phase, but they naturally differed in surface functionality and magnetic properties due to the differences in the properties of the modifier carboxylic acids used and their concentrations. Pure P(VDF-TrFE) and composite scaffolds demonstrate a defect-free morphology and a fiber diameter of about 1 μm. The surface of the P(VDF-TrFE) fibers contains Fe_3_O_4_ nanoparticles, which have a more uniform distribution in the case of Fe_3_O_4_-CA nanoparticles due to the stabilizing effect of the free polar carboxyl groups of CA and their good affinity to the polar solvents used as well as the P(VDF-TrFE) functional groups. In the case of Fe_3_O_4_-OA nanoparticles, stabilization, to a lesser extent, can occur due to a steric hindrance of the bulky alkyl substituent OA and its hydrophobic interactions with low-polarity P(VDF-TrFE) moieties.

The XRD patterns of P(VDF-TrFE) composite scaffolds with Fe_3_O_4_ nanoparticles contain only a magnetite phase, demonstrating that selected electrospinning conditions do not affect the phase composition of both types of Fe_3_O_4_ nanoparticles. The intensity of Fe_3_O_4_ reflections increases proportionally with an increase in the magnetic filler concentration. In this case, the P(VDF-TrFE) composite scaffolds contain a predominantly piezoactive β-phase, along with a small amount of γ-phase, which exhibits weaker piezoelectric properties. The formation of the latter is a consequence of the influence of the electric field, the nature and composition of the solvents used on the behavior, and the rate of P(VDF-TrFE) crystallization. When a magnetic filler was added, no significant decrease in the degree of crystallinity is observed, and for composite scaffolds containing 15 wt.% of both types of Fe_3_O_4_ nanoparticles, the highest degree of crystallinity of about 76% is observed. This result is a consequence of the interaction of CA and OA located on the surfaces of Fe_3_O_4_ nanoparticles with functional groups of P(VDF-TrFE) macromolecules, which promotes their crystallization and, by increasing the total content of the β-phase, improves the piezoelectric properties of magnetoactive scaffolds. The saturation magnetization naturally increases in proportion to the content of the magnetic filler. The highest saturation magnetization value of 10.0 emu/g was obtained for P(VDF-TrFE) scaffolds with 15 wt.% of Fe_3_O_4_-CA nanoparticles. All fabricated composite P(VDF-TrFE) scaffolds exhibit a non-zero coercive force, characterizing their ferromagnetic behavior. An increase in the coercive force in the case of P(VDF-TrFE)/Fe_3_O_4_-OA scaffolds indicates a less uniform distribution of the filler particles and the formation of agglomerates. Based on the obtained experimental data, a scheme of possible interactions between modified Fe_3_O_4_ nanoparticles and P(VDF-TrFE) macromolecules via hydrogen bonding and dipolar and hydrophobic interactions is proposed. The developed magnetoactive composite scaffolds based on P(VDF-TrFE) and modified Fe_3_O_4_ nanoparticles with high saturation magnetization and uniform distribution of magnetic filler should be considered as promising materials for biomedical applications. Moreover, the suggested modified Fe_3_O_4_ nanoparticles, suspension method of their introduction, and electrospinning parameters can be used for the improvement of β-phase content and piezoresponce, not only of P(VDF-TRFE), but also of PVDF.

## Figures and Tables

**Table 1:** Magnetic properties of Fe_3_O_4_-CA and Fe_3_O_4_-OA nanoparticles.

Sample	*M*_s_, emu/g	*M*_r_^a^, emu/g	*H*_c_, Oe
Fe_3_O_4_-CA	61.9 ± 1.9	0.5 ± 0.1	10.0 ± 0.5
Fe_3_O_4_-OA	72.6 ± 3.6	0.6 ± 0.03	8.0 ± 0.4

^a^*M*_r_—remanent magnetization.

**Table 2:** Thermal properties of pure P(VDF-TrFE) and composite scaffolds filled with Fe_3_O_4_-CA and Fe_3_O_4_-OA nanoparticles.

Sample	*T*_m_, °C	Δ*H*_m_, J/g	*X*_c_,%
P(VDF-TrFE)	147.4	27.30	60.7
P(VDF-TrFE)/Fe_3_O_4_-CA 5%	147.7	25.58	59.8
P(VDF-TrFE)/Fe_3_O_4_-CA 10%	147.8	22.80	56.3
P(VDF-TrFE)/Fe_3_O_4_-CA 15%	148.4	29.04	75.9
P(VDF-TrFE)/Fe_3_O_4_-OA 5%	148.2	32.00	74.8
P(VDF-TrFE)/Fe_3_O_4_-OA 10%	148.4	26.68	65.9
P(VDF-TrFE)/Fe_3_O_4_-OA 15%	147.9	29.42	76.9

**Table 3:** Magnetic properties of composite P(VDF-TrFE) scaffolds filled with different contents of Fe_3_O_4_-CA and Fe_3_O_4_-OA nanoparticles.

Sample	*M*_s_, emu/g	*M*_r_^a^, emu/g	*H*_c_, Oe
P(VDF-TrFE)/Fe_3_O_4_-CA (5 wt.%)	4.4 ± 0.2	0.14	28 ± 1
P(VDF-TrFE)/Fe_3_O_4_-CA (10 wt.%)	7.9 ± 0.4	0.18	22 ± 1
P(VDF-TrFE)/Fe_3_O_4_-CA (15 wt.%)	10.0 ± 0.5	0.26	24 ± 1
P(VDF-TrFE)/Fe_3_O_4_-OA (5 wt.%)	4.2 ± 0.2	0.16	35 ± 2
P(VDF-TrFE)/Fe_3_O_4_-OA (10 wt.%)	6.8 ± 0.3	0.23	30 ± 2
P(VDF-TrFE)/Fe_3_O_4_-OA (15 wt.%)	8.8 ± 0.4	0.16	17 ± 1

^a^ M_r_—remanent magnetization.

## Data Availability

The data presented in this study are available on request from the corresponding author.
